# Effect of Electroless Coatings on the Mechanical Properties and Wear Behavior of Oriented Multiwall Carbon Nanotube-Reinforced Copper Matrix Composites

**DOI:** 10.3390/nano11112982

**Published:** 2021-11-06

**Authors:** Zhong Zheng, Jishi Liu, Jiafeng Tao, Jing Li, Wenqian Zhang, Xiuhong Li, Huan Xue

**Affiliations:** School of Mechanical Engineering, Hubei University of Technology, Wuhan 430068, China; jishigod@163.com (J.L.); tjf18717191324@163.com (J.T.); lijing@hbut.edu.cn (J.L.); wenqian_zh@hbut.edu.cn (W.Z.); 20200005@hubt.edu.cn (X.L.)

**Keywords:** multiwall carbon nanotubes, copper composites, electroless coating, wear behavior, mechanical properties

## Abstract

The effects of electroless coatings on the microstructure and composition of the interface between multi-walled carbon nanotubes (MWCNTs) and a Cu matrix and the mechanical properties and wear behavior of the resulting copper matrix composites were investigated. Ni and Cu coatings were electrolessly plated on MWCNTs and mixed subsequently with copper powder. Then copper matrix composites were prepared by sintering, hot extrusion and cold drawing processes. The results showed that MWCNTs were straight, long, uniformly dispersed and aligned in the composites. The Ni coating is more continuous, dense and complete than a Cu coating. The tensile strength, compressive strength, microhardness and tribological properties of Ni@MWCNTs/Cu composite along the drawing direction were enhanced most. The ultimate tensile strength and compressive strength were 381 MPa and 463 MPa, respectively. The friction coefficient and wear rate were reduced by 59% and 77%, respectively, compared with pure Cu samples. This study provides a new insight into the regulation of tribological properties of composites by their interface.

## 1. Introduction

Copper and copper alloys have good conductivity, formability and corrosion resistance, low cost and easy manufacturing. They are widely used in precision gears, precision bearings, high-voltage bearings, high-speed railway grid contact lines/pantographs, slippers/tracks, resistance spot welding electrodes, automotive synchronous gear rings and other key components [1,2]. With the rapid development of precision machinery, high-speed electrified railways, aerospace, automotive and other fields in recent years, the friction and wear conditions on these working surfaces deteriorate rapidly, which leads to a significant decline in the service life and stability of existing parts. One of the effective ways to solve this problem is to select an appropriate reinforcement and control the composite structure and interface in order to prepare copper matrix composites with excellent wear resistance and mechanical properties.

Two types of reinforcements are commonly used to improve the wear resistance of copper in the current industrial field. The first is by adding hard and wear-resistant ceramic particles such as Al_2_O_3_ [3], TiB_2_ [4], B_4_C [5], TiB_2_, SiC [6] and Zr_2_O_3_. For example, the hardness and wear resistance of Al_2_O_3_ dispersion-strengthened copper prepared by an internal oxidation method are greatly improved. However, the internal oxidation process is complex, the production cost is high, and the strength increases at the expense of a loss of ductility and formability, which leads to easy cracking during extrusion and working. The second is by adding solid lubricant reinforcements such as WS_2_, NbSe_2_ [7], graphite fiber and graphite particles [8]. The self-lubricating property of the composites reinforced with solid lubricants make their tribological properties better than those reinforced with ceramic reinforcements [9]. However, the mechanical properties of the latter are reduced [10].

Moreover, some new carbon material reinforcements such as carbon fiber [11], graphene [12] and carbon nanotubes (CNTs) can effectively improve the mechanical and tribological properties of metal matrix composites. Chu et al. [13] arranged graphene nanoplatelets (GNPs) in a copper matrix by vacuum filtration and spark plasma sintering. The results indicated that the oriented arrangement of graphene in the copper matrix contributes to a significant reinforcement in the anisotropic mechanical properties of the composites. CNTs have attracted much attention in the composites field due to their perfect bonding configurations and many excellent properties, including good thermal stability, excellent mechanical properties and axial conductivity [14,15,16,17,18]. More importantly, CNTs are hollow tubes composed of graphite with good self-lubricating properties and are ideal candidates for reinforcements to enhance the mechanical properties and wear resistance of a copper matrix [9,19]. In 2000 Dong et al. [20] first reported the conductivity, mechanical and sliding wear properties of CNT-reinforced copper matrix composites. They prepared copper matrix composites by electroless nickel plating on CNTs, powder metallurgy and rolling. The hardness of the composites is 115~125 HV, and the wear resistance was better than that of CF/Cu. The friction and wear resistance of composites with 8~15 vol.% CNTs was the best. Since then, the mechanical and tribological properties of CNT- reinforced copper matrix composites have been studied [11,21,22]. These studies indicated the excellent tribological properties of CNTs/Cu composites. CNTs/Cu composites were prepared by Tsai et al. [23] through acid treatment, calcination and consolidation technology. A carbon solid lubricant film at the contact interface formed during sliding, and the wear resistance of copper matrix was enhanced by at least 40%. In addition, copper oxide formed on CNTs during calcination and reduction can strengthen the interfacial bonding between the CNTs and the Cu matrix. Akbarpour et al. [24] prepared CNTs/Cu composites by a flake powder metallurgy method. The results demonstrated that the friction and wear properties of Cu-4 vol.% CNT composite are the best. The wear resistance of the composite is improved due to the formation of a CNT-rich film that acts as a solid lubricant layer. Nevertheless, ball-powder-ball collisions can damage or even break CNTs.

According to the published literature, the performance advantages of CNTs have not been fully utilized in the macroscopic properties of CNTs/Cu composites, which cannot currently meet the application requirements proposed by the development of related fields. The mechanical properties and wear behavior of CNTs/Cu composites depend on a variety of internal characteristics, mainly including the amount, spatial distribution and arrangement of the CNTs and the interface between the CNTs and the Cu matrix [25]. That is to say, when CNTs are dispersed in the matrix and arranged along the direction of force/electricity/heat conduction, their excellent mechanical properties, axial conductivity, and extremely high aspect ratio can be effectively utilized. However, van der Waals forces and electrostatic attraction between CNTs make them easily entangled and agglomerated, so it is difficult to disperse and align them [26,27,28,29,30]. In our previous work, CNTs were uniformly distributed and oriented in Cu matrix by nitrogen and a mechanical stirring-assisted ultrasonic dispersion, hot extrusion and cold drawing process. CNTs in the composites maintained their integrity [28]. That method was adopted in this work too.

In addition, the interface between CNTs and Cu matrix is also a key factor determining the mechanical properties and wear behavior of composites. It undertakes the role of transferring load, heat and current between the Cu matrix and CNTs, and blocking any crack propagation. However, the wettability of CNTs on the copper matrix is limited and there is no chemical reaction between Cu and CNTs. Many literatures report that CNTs and Cu matrix are weakly mechanically bonded rather than chemically bonded, resulting in poor mechanical properties of the composites. There are three ways to strengthen interface bonding. The first is acid treatment of CNTs, the introduction of oxygen-containing functional groups on CNTs, and finally the interfacial bonding of Cu-O-C covalent bonds [24]. The second is by adding carbide-forming elements to trigger interfacial reactions and introduce a carbide intermediate layer [31]. The third is by depositing an intermediate metal layer on CNTs [32,33]. However, the first two methods can damage or even consume CNTs and reduce the load transfer capacity of CNTs. The reaction interface between CNTs and carbide-forming elements also introduces brittle impurities, bringing about decreased mechanical and physical properties. The third method does not cause damage to CNTs, so it is considered to be one of the most effective techniques. Previous studies have shown that when Ni or Cu layers were deposited on CNTs as an intermediate layer it established tight bonding between CNTs and the Cu matrix [34,35,36,37], and the CNTs were not damaged [28,38,39].

Various complex coating methods have been applied to enhance the interfacial bonding between CNTs and a Cu matrix, and the mechanical and tribological properties are also improved more or less as a result. The related friction mechanisms are obviously different and are not fully understood. Therefore, the main purpose of this work was to investigate the influences of the spatial distribution and directional arrangement of CNTs, and the composition and structure of the interface on the mechanical properties and wear behavior of the composites. We used an electroless plating method to deposit Ni, Cu and Ni and Cu coatings on multi-wall carbon nanotubes (MWCNTs) as intermediate layers to strengthen the wettability and interfacial bonding between CNTs and the matrix. Then, copper matrix composites were fabricated by mechanical stirring-assisted ultrasonic dispersion, hot extrusion and cold drawing. This study can provide practical and theoretical basis for engineering applications and large-scale industrial preparation of CNTs/Cu composites.

## 2. Experimental

### 2.1. Materials and Methods

The MWCNTs adopted (external diameter 10~20 nm, internal diameter 5~10 nm, length < 30 μm, purity > 90 wt.%, density 0.22 g/cm^3^) were produced by Chinese Academy of Sciences Chengdu Organic Chemistry Co, Ltd. (Chengdu, China). Copper powder with purity of 99.8%, particle size of 45~75 μm and density of 8.905 g/cm^3^ was used.

After sensitization and activation of the MWCNTs [36], electroless nickel plating and copper plating were carried out. The formulas and conditions of the electroless plating are shown in Table 1 and Table 2. Coated MWCNTs (3 vol.%), Cu powder, 0.3 wt.% zinc stearate% zinc stearate and anhydrous ethanol were mixed together and subjected to water bath ultrasonic treatment at 60 °C and 40 kHz, accompanied by stirring until a thick slurry was obtained. The mixture was dried under vacuum at 40 °C for 8 h. Then, following our previously reported process [28], namely sintering, hot extrusion, cold drawing and turning, Φ16 composite bars were obtained. Pure copper and MWCNTs/Cu composite samples were prepared by the same method as a comparison reference.

Here B-MWCN, Ni@MWCN and Cu@MWCNT are used to represent uncoated MWCNT, Ni Coated MWCNT and Cu-coated MWCNT, respectively. B-MWCNTs/Cu composite, Ni@MWCNTs/Cu composite and Cu@MWCNTs/Cu composite denote MWCNTs-reinforced Cu matrix composite, Ni-coated MWCNTs-reinforced Cu matrix composite and Cu-coated MWCNTs-reinforced Cu matrix composite.

### 2.2. Characterization

The microstructure was characterized by field emission scanning electron microscope (FE-SEM, Apreo, FEI, Waltham, MA, USA, Equipped energy dispersion spectrometer(EDS)), transmission electron microscope (TEM, Talos F200X, FEI, Waltham, MA, USA), FIB (Focused Ion Beam)-SEM dual-beam system (Scios 2, FEI, Waltham, MA, USA). Foils for TEM detection were fabricated in vacuum by ion beam thinning instrument (Gatan PIPS, Model 691, Waltham, MA, USA).

The tensile and compression properties of the samples were measured by an Instron3369 universal testing machine (Dongguan hangcheng Electronic Technology, Dongguan, China), with reference to ASTM E9-89a (2000). The tensile specimen is a dog bone with a standard size of 16.5 mm × 6 mm × 2 mm. The cylindrical compressed samples size is standard Φ18 × 25 mm, and its surface roughness Ra < 0.8. The tensile test was carried out at a strain rate of 10^−4^ s^−1^ at room temperature. The results are the average values of five measurements. The microhardness at room temperature was measured by an automatic Vickers hardness tester (HMV-FA2, Shimadzu, Kyoto, Japan). The sample was mechanically polished. The load was 0.49 N, and the residence time was 15 s. The microhardness data are the average of 10 measurements.

A UNT-Tribolab (Bruker, Bremen, Germany) friction and wear tester was used for dry sliding wear test to avoid the influence of lubricants such as lubricating oil or grease. The sample size was 15 × 15 × 5 mm. The surface was ground and polished with silicon carbide paper, and the surface roughness was Ra < 0.8. The 440 carbon steel balls with a diameter of 10 mm and hardness of 62 HRC were used as counter friction pair. The dry sliding wear test was carried out at 20 ± 5 °C and relative humidity of 50 ± 5%. The applied load was 25 N, the sliding speed was 10 mm/s, and the reciprocating stroke was 1000 times. The results are the average values of five measurements. The wear rate was calculated by the following formula:

(1) $$W=\frac{V}{L\cdot D}$$

where *W*, *V*, *L* and *D* are the wear rate (mm^3^·N^−1^·m^−1^), wear amount (mm^3^), normal load (N) and sliding distance (m), respectively.

## 3. Results and Discussions

### 3.1. Microstructure

**Figure 1:**
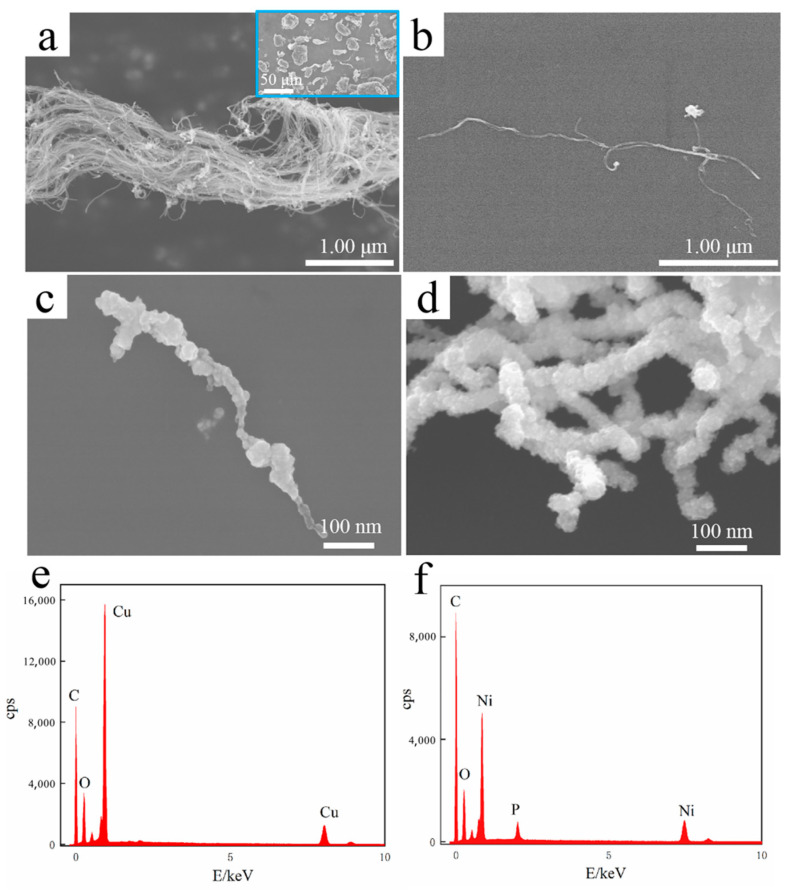
SEM micrographs of (**a**) original MWCNTs, (**b**) MWCNT after dispersion treatment, (**c**) Cu@MWCNTs, (**d**) Ni@MWCNTs, EDS of (**e**) Cu@MWCNT, (**f**) Ni@MWCNT.

Figure 1 shows SEM micrographs of original and electroless plated MWCNTs. The original MWCNTs were entangled into bundles of ~1 μm in diameter and aggregated into ~30 μm particles (Figure 1a). MWCNTs were dispersed into single units by ultrasonic treatment, and no damage or interruption is found (Figure 1b). EDS (Figure 1e,f) indicates that the surface of MWCNTs (Figure 1c,d) is electrolessly plated. However, the Ni coating (Figure 1d) was more complete, uniform and continuous than the Cu coating (Figure 1c). This is because Pd catalyst particles were attached to sensitized MWCNTs during electroless plating, and Ni was reduced and deposited on them. After the Pd catalyst particles were completely covered, the reduction reaction of Ni can continue due to the self-catalytic activity of Ni, filling the gap between the plating areas or increasing the thickness of coating, and finally forming a complete, uniform and continuous coating. The potential of Cu without catalytic activity is more positive than that of Ni. Once the Pd catalyst particles were completely covered, the reduction reaction of Cu stopped immediately, give rise to incomplete and uniform copper coating.

**Figure 2:**
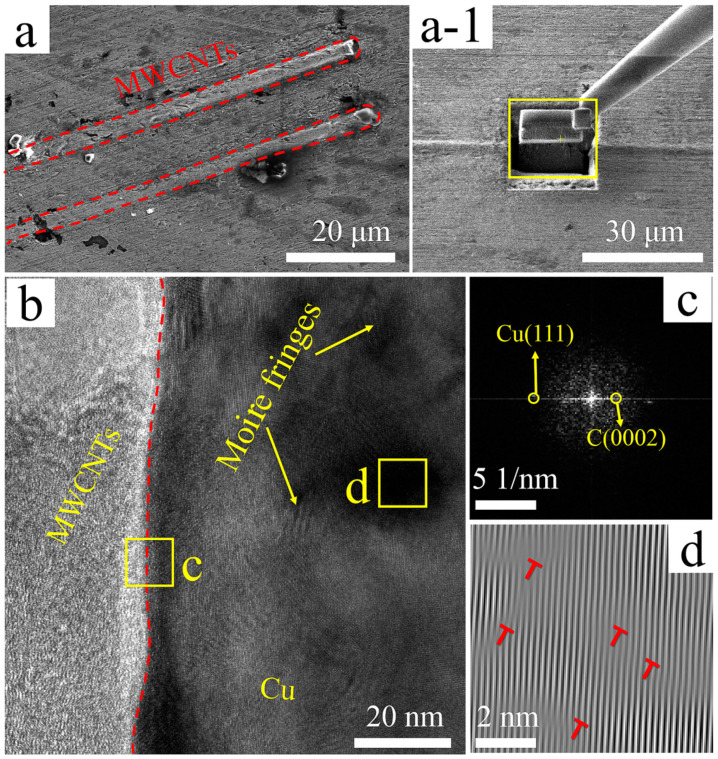
(**a**) SEM micrograph of B-MWCNTs/Cu composite, (**a-1**) Sampling the manipulator at a, (**b**) TEM micrograph, (**c**) FFT image of region c in b, (**d**) IFFT image of region d in b, dislocations marked with ‘T’ symbols.

**Figure 3:**
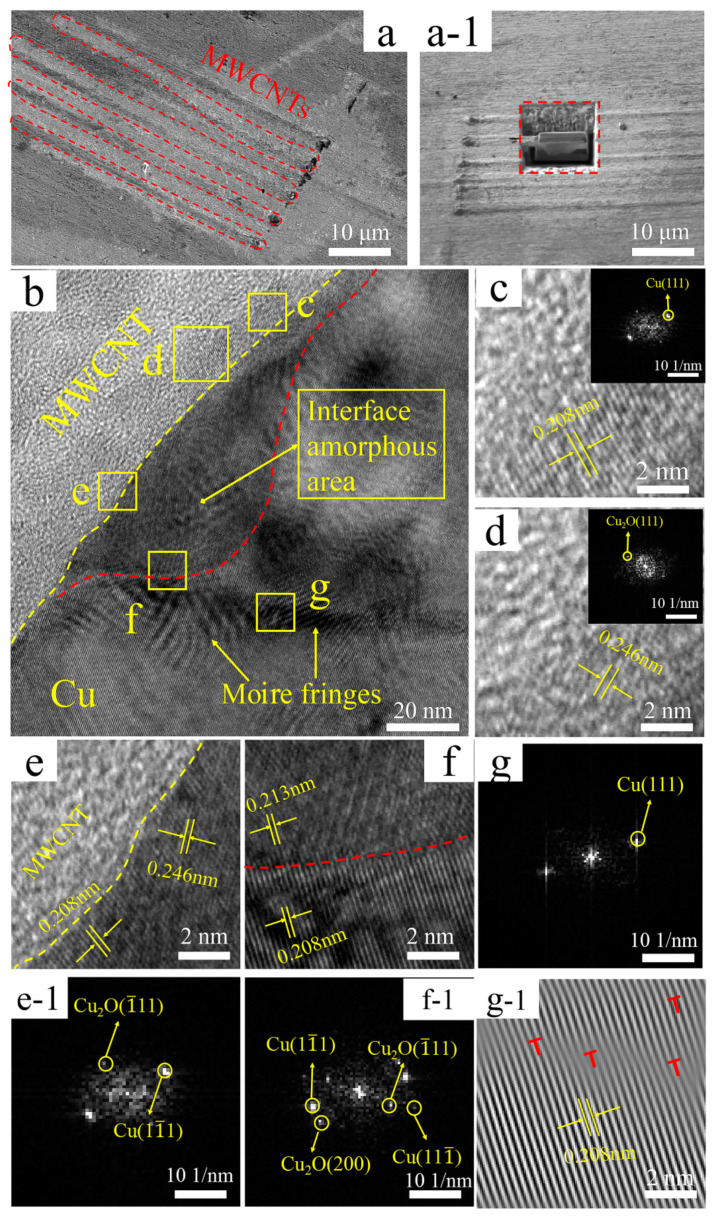
(**a**) SEM micrograph of Cu@MWCNTs/Cu composite, (**a-1**) Sampling the manipulator at a, (**b**) TEM micrograph, (**c**) Enlarged view and FFT image of region c in b, (**d**) Enlarged view and FFT image of region d in b, (**e**,**e-1**) Enlarged view and FFT image of region e in b, (**f**,**f-1**) Enlarged view and FFT image of region f in b, (**g**,**g-1**) FFT and IFFT image of region g in b, dislocations marked with ‘T’ symbols.

**Figure 4:**
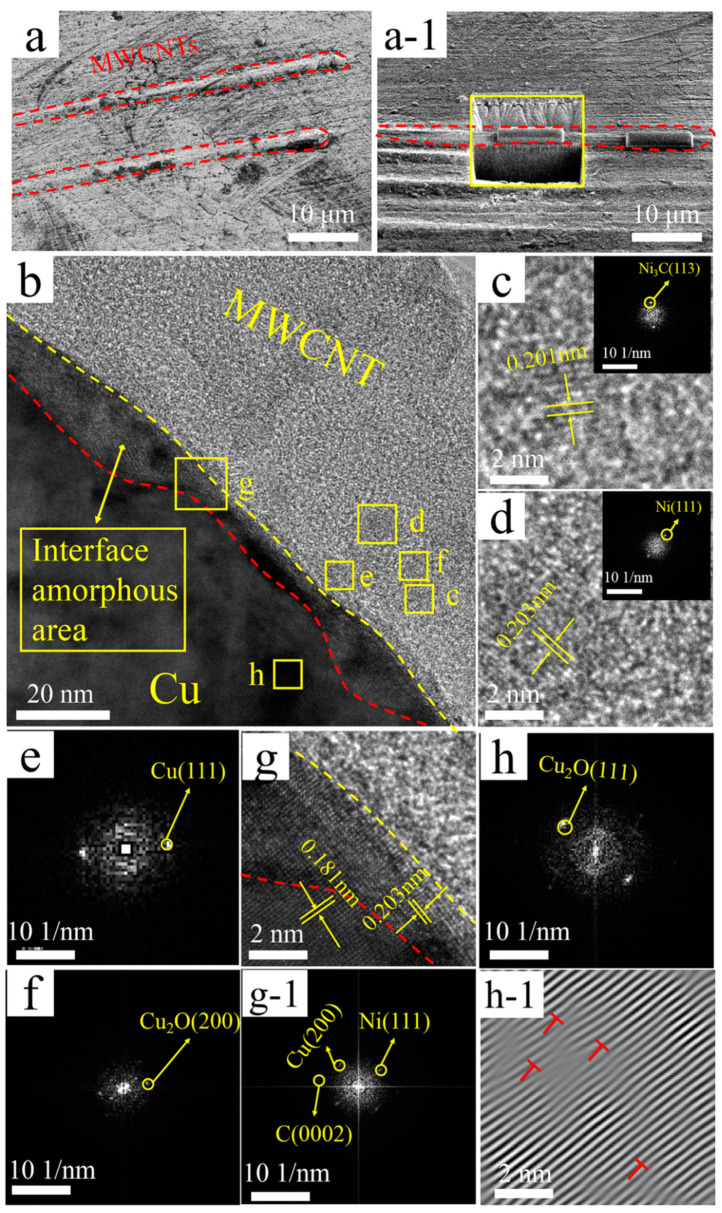
(**a**) SEM micrograph of Ni@MWCNTs/Cu composite, (**a-1**) Sampling the manipulator at a, (**b**) TEM micrograph, (**c**) Enlarged view and FFT image of region c in b, (**d**) Enlarged view and FFT image of region d in b, (**e**) FFT image of region e in b, (**f**) FFT image of region f in b, (**g**,**g-1**) Enlarged view and FFT image of region g in b, (**h**,**h-1**) FFT and IFFT images of region h in b, dislocations marked with ‘T’ symbols.

Figure 2 shows SEM and TEM micrographs of B-MWCNTs/Cu composite samples. Many directional long rod-like protrusions can be seen in Figure 2a. Ultra-thin sections with thickness of ~0.1 μm were obtained by FIB cutting along rod-like protrusions, as shown in Figure 2(a-1). The thinned slices were used for TEM characterization, and it can be confirmed that the protrusions were MWCNTs. These MWCNTs are straight, long, separated, parallel to each other and arranged in the same direction. Similar long rod-like protrusions can also be found on the surface of Cu@MWCNTs/Cu composite (Figure 3a) and Ni@MWCNTs/Cu composite (Figure 4a). This is due to the high stress and strain generated during hot extrusion and drawing, which prompted MWCNTs to be straightened and reoriented along the direction of extrusion and drawing.

From the TEM micrograph of the interface region in Figure 2b and the corresponding fast Fourier transform (FFT) image in Figure 2c, it can be inferred that the left side of the red dotted line in Figure 2b corresponds to the graphite structure C(0002) lattice of MWCNTs, and the right side is the Cu(111) lattice. There is no transition or gap between them. Meanwhile, the crystal planes on both sides of the red dotted line are not parallel, indicating that MWCNTs and Cu matrix were mechanically attached. There are many darker color regions on the right side of red dotted line. Figure 2d is the corresponding inverse fast Fourier transform (IFFT). The Moire fringe shows the existence of dislocations (‘T’ symbols). The dislocations originated from the severe deformation of hot extrusion and drawing, which generated large compressive stress and shear strain in the interface areas.

It can be deduced from the TEM and the corresponding FFT images of the interface region of Cu@MWCNTs/Cu composite in Figure 3b that the upper left side of yellow dotted line is the graphite structure C(0002) lattice of MWCNTs, an amorphous region is between the red and yellow dotted lines, and the lower right side of the red dotted line is the Cu matrix. As shown in Figure 3c–e (region c~e in Figure 3b), Cu(1$\bar{1}$1) plane, Cu(11$\bar{1}$) plane, Cu_2_O($\bar{1}$11) plane, Cu_2_O(200) plane are detected, and (0002) _MWCNT_//($\bar{1}$11)_Cu_, indicating that these are electroless copper coatings formed in situ on MWCNTs. After the mixture of Cu@MWCNTs and copper powder was sintered and hot extruded, amorphous zones were formed between MWCNTs and copper matrix. As shown in Figure 3f (region f in Figure 3b), at the junction of amorphous region and Cu matrix, Cu(111) plane, Cu(1$\bar{1}$1) plane, Cu_2_O(111) plane, and Cu_2_O($\bar{1}$11) plane were detected, and (200)_Cu2O_//($\bar{1}$11)_Cu_, indicating the formation of coherent interface between them.

All these indicate that tight bonding ocurred between MWCNTs, amorphous regions and Cu matrix. In addition, the copper coating and amorphous regions indicate anisotropic morphology and a polycrystalline structure, which also helps to stabilize the interface structure [23]. Cu_2_O may be formed by oxidation of electroless Cu coating during powder mixing and sintering.

Dislocations are also found in Cu matrix in Figure 3(g-1) (region g-1 in Figure 3b) (‘T’ symbol), where the dislocation density is higher than that in B-MWCNTs/Cu composite. These dislocations are partly due to the compressive stress and shear strain produced by severe deformation during hot extrusion and drawing, partly due to the lattice distortion caused by the different lattice parameters of Cu_2_O and Cu, and some are due to the different thermal expansion coefficients of MWCNTs and Cu [40,41]. During the cooling process after high temperature treatment such as sintering and hot extrusion, dislocations were generated at the interface [42].

From the TEM and the corresponding FFT images of Ni@MWCNTs/Cu composite in Figure 4b, it can be concluded that the upper right side of yellow dashed line in Figure 4b is the graphite structure C(0002) lattice of MWCNTs, the amorphous region is between the red and yellow lines, and the left lower side of red line is the Cu matrix. As shown in Figure 4c–f (region c~f in Figure 4b), Ni_3_C(113) plane, Ni_3_C($\bar{1}$10$\bar{1}$) plane, Ni(111) plane, Cu(111) plane, Cu_2_O(200) plane are detected on MWCNTs. Ni_3_C was formed by the reaction of carbon atoms from MWCNTs with electroless Ni coating during sintering and hot extrusion. As shown in Figure 4g (region g in Figure 4b), the C(0002) lattice of MWCNTs gradually transits from the amorphous region to the Cu(200) lattice of the Cu matrix. As shown in Figure 4(g-1) (region g-1 in Figure 4b), Ni(111) plane and Cu(200) plane are detected in amorphous region, which are solid solutions of Cu and Ni. The binary phase diagram of Cu/Ni indicates that Cu and Ni can form all proportions of solid solution. Their face-centered cubic structures are the same, and their thermophysical properties are similar, so the interface bonding can be effectively enhanced [15,16]. As shown in Figure 4(h-1) (region h-1 in Figure 4b), the dislocation density in Cu matrix is higher than that in B-MWCNTs/Cu composite and Cu@MWCNTs/Cu composite. The dislocation movements can be suppressed more effectively, thereby reinforcing the MWCNT/Cu composite.

### 3.2. Mechanical Properties

**Figure 5:**
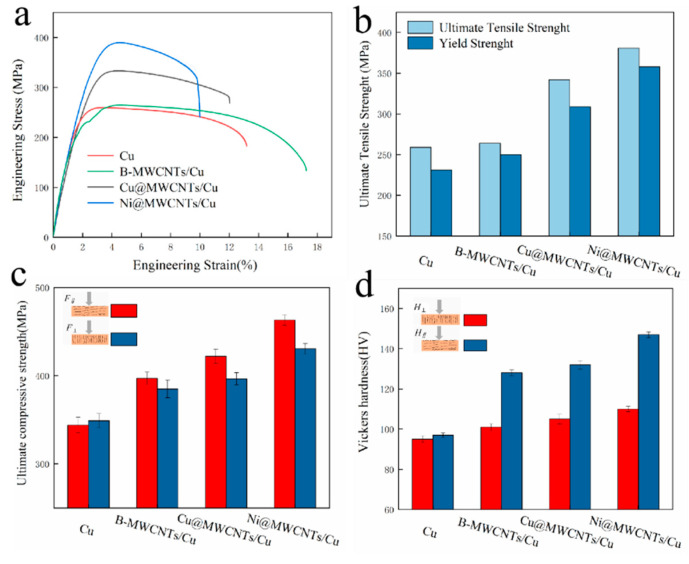
Mechanical properties of pure Cu and MWCNTs/Cu composites. (**a**) Stress-train curve, tensile test direction parallel to drawing direction. (**b**) UTS and YS. (**c**) Compressive strength. *F*_∥_: test surface was parallel to drawing direction. *F*_⊥_: test surface was perpendicular to drawing direction. (**d**) Vickers microhardness. *H*_∥_: test surface was parallel to drawing direction, *H*_⊥_: test surface was perpendicular to drawing direction.

Figure 5a,b show the stress-strain curves, ultimate tensile strength (UTS) and yield strength (YS) of pure Cu and MWCNTs/Cu composite samples. The tensile test direction was parallel to the drawing direction. Figure 5c,d show the compressive strength and Vickers microhardness of pure Cu and MWCNTs/Cu composites samples on different test planes. The results indicates that the UTS, YS, compressive strength and microhardness of MWCNTs/Cu composites are higher than those of pure Cu. The reason is that the Young’s modulus of MWCNTs (about 270–950 GPa [43]) is much higher than that of copper (117 GPa), which reduces the deformation or damage caused by the test load. In addition, the UTS, YS, compressive strength and microhardness of the composites from high to low are Ni@MWCNTs/Cu composite > Cu@MWCNTs/Cu composite > B-MWCNTs/Cu composite. For example, the mechanical properties of Ni@MWCNTs/Cu composite sample on the test plane/direction parallel to the drawing direction are the best. The UTS, YS, compressive strength and microhardness are 381 MPa, 358 MPa, 463 MPa and 147 HV, respectively. Compared with the B-MWCNTs/Cu composite sample on the same plane, the UTS, YS, compressive strength and microhardness increased by 44.3%, 43.2%, 16.7% and 14.8%, respectively. Compared with pure Cu sample on the same plane, the UTS, YS, compressive strength and microhardness increased by 47.1%, 51.9%, 34.5% and 51.5%, respectively.

The nanophase strengthening mechanism of metal matrix composites generally includes a load transfer effect from the matrix to reinforcements [44], grain refinement [45], Orowan ring strengthening [46]. We proved that the enhancement of mechanical properties of MWCNTs/Cu composites was mainly attributed to load transfer effect from matrix to MWCNTs in our previous studies [28]. It can be seen from Figure 2a and Figure 3a that MWCNTs are aligned along the load direction of tensile test. Therefore, the following modified shear lag formula can be used to calculate the theoretical yield strength of composite materials [47]:

(2) $$l_{c}=\frac{\sigma_{CNT}d}{2\tau_{m}}=3.085\;\mu m$$

(3) $$\sigma_{c}=\sigma_{m}\left(1-V_{CNT}\right)+\sigma_{CNT}V_{CNT}\left(1-\frac{l}{2l_{c}}\right)\;\;\;\;\mathrm{for}\;l>l_{c}$$

where $l_{c}$ is the critical length, and l is the length of MWCNT(~5.5 μm);$\;\sigma_{c}$, $\sigma_{m}$, $\sigma_{CNT}$ is the yield strength of composite material; matrix and MWCNT (~39 GPa) respectively;$\;V_{CNT}$ is the volume fraction of MWCNTs (3 vol.%); d is the diameter of MWCNTs (~20 nm); $\tau_{m}$ is the yield shear strength of Cu matrix ($\tau_{m}=1$/2$\sigma_{m}$). The calculated theoretical yield strength of 3 vol.% MWCNTs-reinforced Cu matrix composites is 395.14 Mpa. It accounted for 94.91%, 86.07%, and 64.57% of the actual measured values of Ni@MWCNTs/Cu composite, Cu@MWCNTs/Cu composite, and B-MWCNTs/Cu composite samples, respectively, indicating that the ranking of the load transfer effect in the reinforcement mechanism of MWCNTs/Cu composites from large to small is Ni@MWCNTs/Cu composite > Cu@MWCNTs/Cu composite > B-MWCNTs/Cu composite. The interface between MWCNTs and matrix undertook the role of transferring load from matrix to MWCNTs to block crack propagation. Therefore, the interface was the main contribution to the mechanical properties of these composites. According to the analysis of the interface of each composite sample in Section 3.2, the wettability between MWCNTs and Cu matrix was strengthened by the electroless coating of MWCNTs, and the chemical bond between MWCNTs and Cu matrix was strong. Compared with the interface between Cu@MWCNTs and Cu matrix, the interface composition, structure and interface bonding state between Ni@MWCNTs and Cu matrix are more conducive to load transfer. The load applied during mechanical performance test can be more effectively transferred from copper matrix to the stronger MWCNTs, so that the MWCNT/Cu composites can be more enhanced. In addition, the density of dislocations stored in the amorphous regions at the interface between Ni@MWCNTs and Cu matrix was also greater, which can more effectively inhibit the movement of dislocations and enhance the composites. In B-MWCNTs/Cu composite sample, the interface between MWCNTs and Cu matrix was only weak mechanical bonding, which cannot effectively transfer loads and hinder the movement of dislocations. Therefore, its tensile strength is the weakest.

The mechanical properties of MWCNTs/Cu composite samples all demonstrate orthotropic anisotropy. The compressive strength and Vickers microhardness on the test planes parallel to drawing direction are significantly higher than those perpendicular to drawing direction. This is due to that MWCNTs are aligned in Cu matrix. When the test load was perpendicular to the arrangement direction of MWCNTs, the excellent axial mechanical properties of MWCNTs can be more effectively used to resist shear stress than that when the test load parallel to the arrangement direction of MWCNTs.

**Figure 6:**
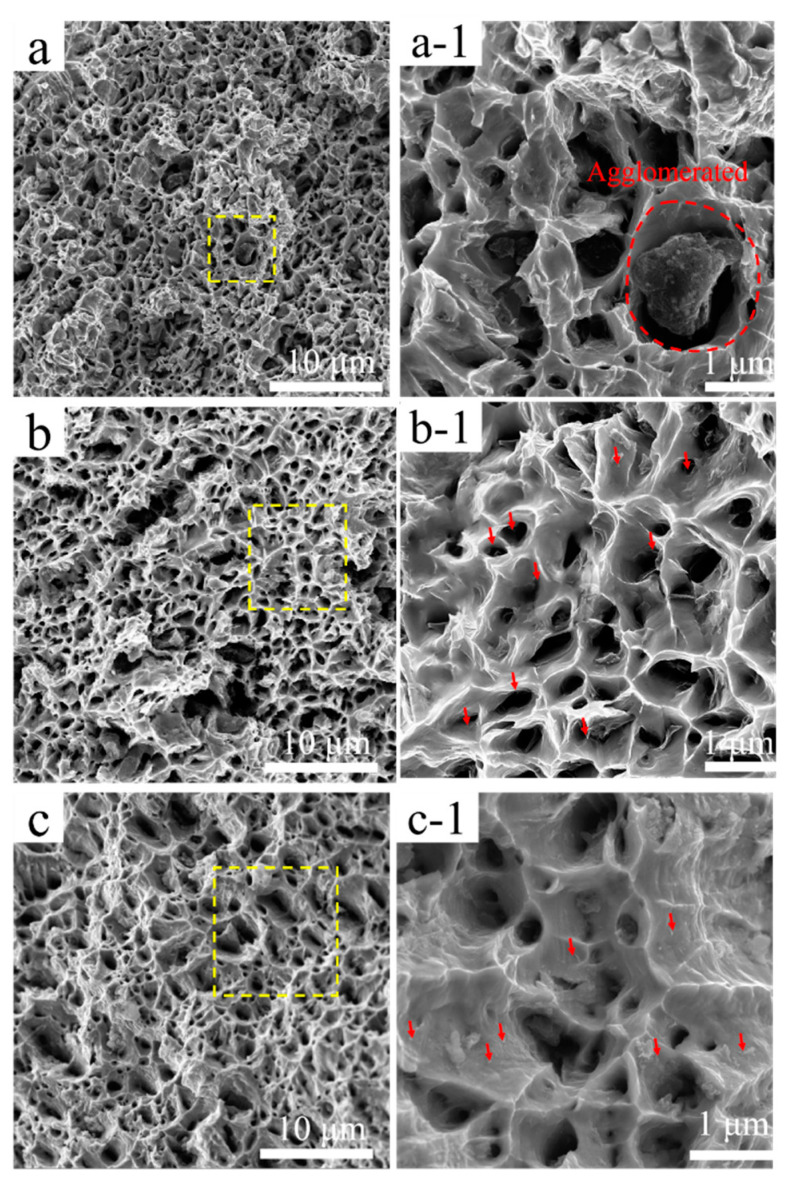
SEM images of tensile fracture of (**a**,**a-1**) B-MWCNTs/Cu composite, (**b**,**b-1**) Cu@MWCNTs/Cu composite, (**c**,**c-1**) Ni@MWCNTs/Cu composite, red arrows marks MWCNTs.

Figure 6 shows SEM images of the tensile fracture of the composite specimens. The fracture surface of the composites exhibits typical plastic fracture characteristics, and uniform dimples with a size of about 1~3 μm. Figure 6a shows the fracture surface of B-MWCNTs/Cu composite, and some scattered irregular large cavities can be seen, with sizes ranging from several to dozens of μm. Agglomerated MWCNTs can be seen in the enlarged images of these large cavities. This indicates that the van der Waals force and electrostatic attraction between B-MWCNTs are more likely to cause entanglement and agglomeration, so that MWCNTs cannot offer any advantage. The voids are also discovered on the fracture surface of Cu@MWCNTs/Cu composite sample, but the number and size are much smaller. This may be because Cu coating on MWCNTs was not complete and uniform, generating a small amount of entanglement and agglomeration. 

On the fracture surface of Ni@MWCNTs/Cu composite sample, only a few cavities with a size of ~5 μm and a small amount of agglomerated Ni@MWCNTs are found. This indicated that there was still more or less MWCNTs agglomeration in Cu matrix.

Many pulled MWCNTs short ends (indicated by red arrows) can be seen on the fracture surfaces of Ni@MWCNTs/Cu composite and Cu@MWCNTs/Cu composite, which are mostly distributed on the tearing ridges, indicating that MWCNTs bore tensile stress or hindered crack propagation before sample fracture during the tensile tests. In addition, some pulled MWCNTs short ends also had metal coatings. All these proved that the interface between MWCNTs and copper matrix is very strong. Some pulled-out MWCNTs short ends are distributed outside the tear ridges in Cu@MWCNTs/Cu composite samples, indicating less load was transferred. This may be because the Cu coating on MWCNTs is not complete and uniform, resulting in weak interface bonding. The pulled-out MWCNTs form long outcrops on the fracture surface of B-MWCNTs/Cu composite, which are significantly less than the coated-MWCNTs/Cu composites. It proves that the interface between MWCNTs and Cu matrix is only a weak mechanical bonding, so that the tensile load cannot be effectively transferred, and MWCNTs can be pulled out from Cu matrix with a small tensile force during the tensile tests.

### 3.3. Frictional Behavior

**Figure 7:**
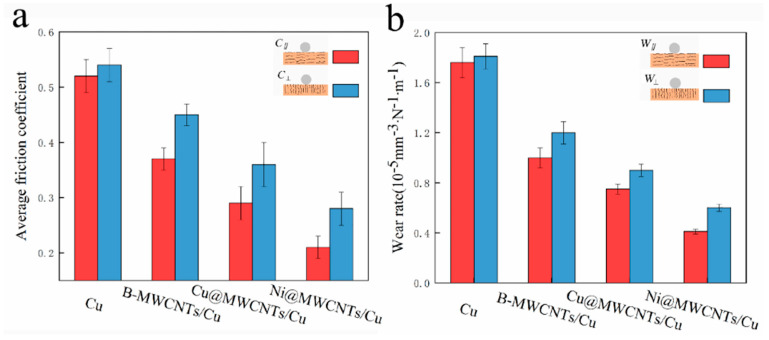
(**a**) Average COF, *C*_∥_: test plane parallel to drawing direction, *C*_⊥_: test plane perpendicular to drawing direction. (**b**) Wear rate, *W*_∥_: test plane parallel to drawing direction, *W*_⊥_: test plane perpendicular to drawing direction.

Figure 7 exhibits the average coefficient of friction (C OF) and wear rate on the two orthogonal friction test surfaces of pure Cu and MWCNTs/Cu composites. It can be seen from Figure 7a that the friction coefficient *C*_∥_ (the friction test surface is parallel to the drawing direction) of MWCNTs/Cu composite samples is far lower than the friction coefficient *C*_⊥_ (the friction test surface was perpendicular to drawing direction). For example, the *C*_∥_ of Ni@MWCNTs/Cu composite sample is 33% lower than that of *C*_⊥_, and its *W*_∥_ is 36% lower than that of *W*_⊥_. Similarly, the wear rate *W*_∥_ (the friction test surface is parallel to drawing direction) is also far lower than the wear rate *W*_⊥_ (the friction test surface is perpendicular to drawing direction), as shown in Figure 7b. The friction coefficient and wear rate of Ni@MWCNTs/Cu composite are the lowest. The friction coefficients *C*_∥_ and *C*_⊥_ are 0.21 and 0.28, respectively, which are reduced by 59% and 48% respectively compared with pure Cu sample. The wear rates *W*_∥_ and *W*_⊥_ are 0.41 × 10^−5^ mm^−3^·N^−1^·m^−1^ and 0.6 × 10^−5^ mm^−3^·N^−1^·m^−1^, respectively, which are 77% and 66% lower than those of pure Cu samples.

**Figure 8:**
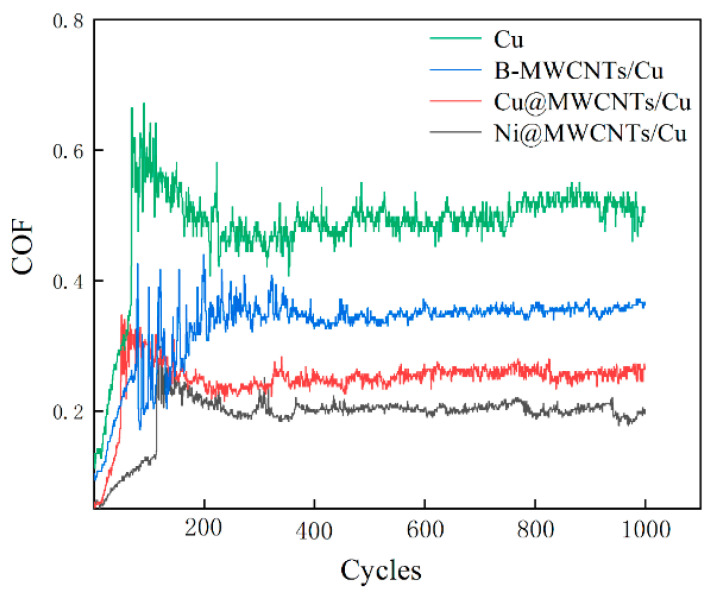
Variation of *C*_∥_ as a function of test cycles (*C*_∥_: Average COF on test plane parallel to drawing direction).

In practical applications, the friction working surface needs to be consistent with the surface with the strongest wear resistance. Therefore, here we are more concerned about the tribological properties of each samples on the surface parallel to the drawing direction. To this end, the COF of the samples on the test surface parallel to drawing direction is plotted as a function of the number of linear reciprocating sliding cycles, as shown in Figure 8.

There is little difference between *C*_∥_ and *C*_⊥_, *W*_∥_ and *W*_⊥_ of pure Cu sample. This indicates that although the hardness on the friction test surface perpendicular to the drawing direction was slightly higher than that parallel to the drawing direction, its effect on reducing friction seems to be small. The *C*_∥_ of pure Cu sample increases from 0.1268 to 0.6645 in about 70 cycles, as shown in Figure 8. This period belongs to the running-in period. At this time, the counter friction ball only contacts with the higher and small bumps on the sample surface, so the contact area was small. With the increase of the number of sliding friction cycles, the higher micro-bulges were worn, the initial surface roughness decreased gradually, and the actual contact area increased. The contact conditions between the counter ball and sample surface were changed from non-conformal to conformal, generating a slight decrease of COF to 0.4751 in about 160 subsequent cycles. It remained stable and reached equilibrium steady state after about 370 cycles.

For B-MWCNTs/Cu composite, *C*_∥_ increased from 0.0943 to 0.426 in the first 70 cycles. In the next 260 cycles, *C*_∥_ fluctuated sharply. After about 330 cycles, *C*_∥_ fluctuated between 0.35 and 0.37. The first increase can be interpreted as a run-in period. Subsequently, the sharp fluctuation of COF is due to the release of MWCNTs to the contact area between the ball and sample surface after the wear of Cu matrix. Due to the uneven distribution of MWCNTs in Cu matrix, there were many clusters. During the wear processing, the unevenly distributed and new MWCNTs clusters were continuously released from Cu matrix to contact areas, providing new solid lubricant. Due to the irregular existence of MWCNTs, the number of MWCNTs transferred to the contact areas varied with time, resulting in dramatic fluctuations in COF.

For Ni@MWCNTs/Cu composite and Cu@MWCNTs/Cu composite samples, the process was similar to that of B-MWCNTs/Cu composite sample. However, during the first increase of *C*_∥_ in running-in period, *C*_∥_ was much smaller than pure Cu and B-MWCNTs/Cu composite, and the cycle number of Cu@MWCNTs/Cu composite sample was also much less, only about 45 cycles. This is mainly due to the strong interfacial bonding between coated MWCNTs and Cu matrix, which facilitated the transfer of the load applied to the ball from copper matrix to the stronger MWCNTs, so as to protect the Cu matrix from damage. During the polishing process of the friction test sample preparation, the softer Cu matrix was first removed from the surface by wear. The stronger MWCNTs can effectively resist shear stress, firmly pinned on the surface parallel to the drawing direction, forming long rod-like protrusions, as shown in Figure 2a, Figure 3a and Figure 4a. These long rod-like protrusions are stronger than Cu matrix and have greater resistance to ploughing of the counter ball, which reduced COF during running-in period. In the third part of the equilibrium steady-state period, the fluctuation of *C*_∥_ was also much smaller than that of B-MWCNTs/Cu composite. This is due to the coating isolated MWCNTs from each other, reducing entanglement and agglomeration. In addition, the coating can also reduce the density difference between MWCNTs and Cu powder, which is helpful to the uniform mixing of powder, so that MWCNTs are uniformly distributed in Cu matrix and the clusters are much smaller. During the wear processing, the amount of solid lubricant MWCNTs released to contact areas was much more regular and stable. The *C*_∥_ and wear rate of Ni@MWCNTs/Cu composite were lower than that of the Cu@MWCNTs/Cu composite, and the fluctuation of *C*_∥_ in the third part of the equilibrium steady state was also smaller. The reason is that Ni coating on MWCNTs is more complete, uniform and continuous than the Cu coating, which contributes to stronger interfacial bonding between MWCNTs and matrix, more effective shear stress resistance, more uniform dispersion and smaller clusters of MWCNTs.

The *C*_∥_ and *W*_∥_ of MWCNTs/Cu composite samples are much lower than *C*_⊥_ and *W*_⊥_. This is mainly owing to the hot extrusion and drawing process made the MWCNTs arranged along the direction parallel to the drawing direction. When the counter ball rubbed on the test surface parallel to the drawing direction, once the coating and outer tubes of MWCNTs were destroyed, the inner tubes fell off and release to contact areas, providing more new solid lubricants. On the sample surface perpendicular to the drawing direction, the counter ball was only radially cut MWCNTs point by point, and the carbon solid lubricant released to contact areas was limited.

### 3.4. Wear Behavior

**Figure 9:**
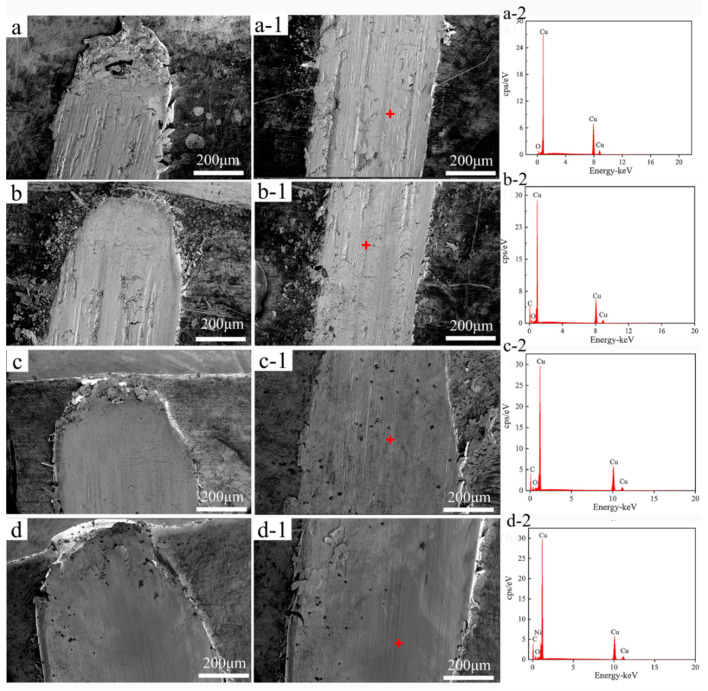
SEM micrographs and corresponding EDS of wear trajectories on the surface parallel to drawing direction of (**a**,**a-1**,**a-2**) Pure Cu, (**b**,**b-1**,**b-2**) B-MWCNTs/Cu composite, (**c**,**c-1**,**c-2**) Cu@MWCNTs/Cu composite, (**d**,**d-1**,**d-2**) Ni@MWCNTs/Cu composite.

**Figure 10:**
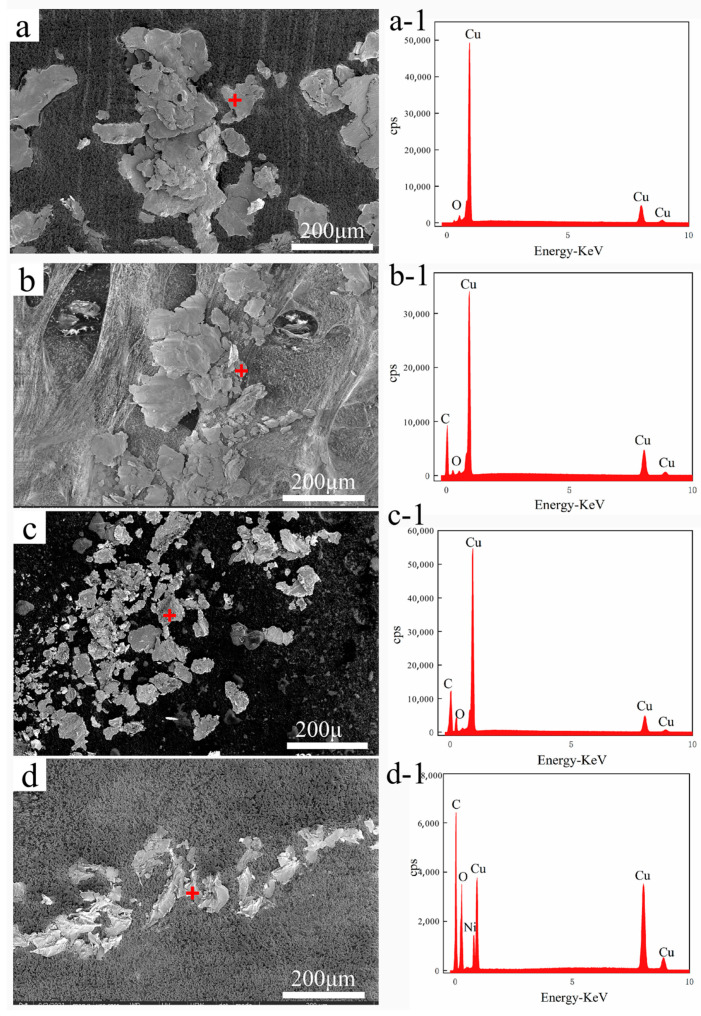
SEM micrograph and corresponding EDS of wear debris collected on the surface parallel to drawing direction of (**a**,**a-1**) Pure Cu, (**b**,**b-1**) B-MWCNTs/Cu composite, (**c**,**c-1**) Cu@MWCNTs/Cu composite, (**d**,**d-1**) Ni@MWCNTs/Cu composite.

Figure 9 shows SEM micrographs and the corresponding EDS analysis of the surface wear trajectories parallel to the drawing direction of pure Cu and MWCNTs/Cu composite samples. Figure 10 exhibits SEM micrographs and EDS analysis of wear debris collected in the wear trajectories.

Tear cracks and spalling pits can be found in the wear trajectory of pure Cu samples (Figure 9(a-1)). The plough grooves are deep. There is a large amount of spalling debris accumulation at the end of the wear trajectory (Figure 9a), which is a typical feature of adhesive wear. The reason is that the yield stress of pure Cu is low, and the hardness of the counter (440 carbon steel ball) is much higher than that of pure Cu. Micro-cutting occurred on the surface of pure Cu sample under the shear stress generated by the sliding between them [48]. As shown in Figure 10a, the wear debris collected in the wear trajectory of pure Cu sample exist in the form of lamellar debris, accompanied by wear cracks. Their size is the largest among all samples, about 30~100 μm. These wear debris have low hardness and good ductility, so plastic deformation took place under normal load generated by sliding, become thin slices, and ‘cold welding’ occurred. Or they were pushed out of contact areas and accumulated at the end of the wear trajectory. Furthermore, the wear trajectory of pure copper sample and the ESD of its wear debris reveal the formation of copper oxide (Figure 9(a-2) and Figure 10(a-1)). The heat generated by sliding friction accelerated the oxidation of the sample surface and wear debris. Under the combined action of normal load and shear stress caused by sliding, the surface copper oxide layer was prone to brittle cracks and tearing. The debris may be composed of a mixture of Cu_2_O, CuO and Cu [49]. Third-body friction caused by wear debris and hard particles [50] formed plough grooves on the worn surfaces.

Tear cracks and spalling pits are also found in the wear trajectory of B-MWCNTs/Cu composite sample (Figure 9(b-1)). The plowings are however very uneven, in some parts of plow grooves are deeper, and in other parts they are flatter. The debris accumulated at the end of the wear trajectory (Figure 9b) is much less than that of a pure Cu sample. As shown in Figure 10b, the wear debris are slightly smaller than that of pure Cu sample. In addition, the wear trajectory of B-MWCNTs/Cu composite sample and the ESD of wear debris demonstrate carbon peaks (Figure 9(b-2) and Figure 10(b-1)). Compared with pure Cu sample, the decrease of friction coefficient and the improvement of wear resistance of B-MWCNTs/Cu composite can be put down to the effect of solid lubricant isolation layer of MWCNTs. During the friction test, MWCNTs were separated from Cu matrix to minimize the direct contact between the counter surfaces [9,51]. The non-uniform wear of its surface is due to the uneven distribution of MWCNTs in Cu matrix, mostly in the form of clusters, contributing to uneven distribution of lubricating graphite friction layer.

For Ni@MWCNTs/Cu composite and Cu@MWCNTs/Cu composite samples, the wear trajectories (Figure 9(c-1),(d-1)) are significantly ameliorated compared with the former two, which are relatively flat. There are no obvious plowing deep grooves, and only uniform distribution of shallow scratches. Moreover, their wear trajectories and debris ESD also indicate the presence of carbon peaks.

The debris at the end of the wear trajectory (Figure 9c,d) accumulate less. As shown in Figure 10c,d, the wear debris are much smaller, about 2~10 μm. This is because the coating made the interfacial bonding between MWCNTs and Cu matrix stronger, and normal load and friction shear stress generated by sliding resistance are more effective, reducing the brittle cracks, tearing and peeling on the surface. Furthermore, MWCNTs are dispersed more uniformly in the Cu matrix, and the solid lubricant MWCNTs released to contact areas are more uniformly distributed.

## 4. Conclusions

Here we have investigated the effect of electroless Ni and Cu coatings on MWCNTs/Cu composites interface to reveal the mechanism of alignment of MWCNTs, microstructure and composition of interface on mechanical properties and wear behavior of MWCNTs/Cu composites.

(1) There are amorphous regions, high density dislocations and the interface products of Cu_2_O and Ni_3_C, Cu and Ni solid solutions in the interface regions of coated-MWCNTs/Cu composites. A strong interface bonding was formed, so the tensile strength, compressive strength, microhardness and tribological properties of the composites were effectively improved. The interface between MWCNTs and matrix in B-MWCNTs/Cu composite is weak mechanical bonding, resulting in poor performance.
(2) The Ni coating is the densest, continuous and complete, forming strong interfacial bonding between MWCNTs and matrix. Therefore, the enhancement of the composites is also the largest. The UTS, YS, compressive strength and microhardness of Ni@MWCNTs/Cu composite along the drawing direction are 381 MPa, 358 Mpa, 463 Mpa and 147 HV, respectively, which are 47.1%, 51.9%, 34.5% and 51.5% higher than those of pure Cu. The COF and wear rate are 0.21 and 0.41 × 10^−5^ mm^−3^·N^−1^·m^−1^, respectively, which are 59% and 77% lower than those of pure Cu, respectively.
(3) The enhancement of wear resistance of composites is attributed to the role of MWCNTs as a solid lubricant isolation layer between the counter surfaces. Due to the electroless coating, the interface bonding is stronger, and MWCNTs are more effective in resisting the normal load and shear stress caused by sliding, thus reducing the brittle cracks, tearing and peeling of the surface layer.
(4) MWCNTs are straight, long, uniform dispersion and aligned in composites. As a consequence, the mechanical and tribological properties of composites exhibit orthogonal anisotropy. When the counter ball rubbed on the test surface parallel to the drawing direction, once the coating and outer tubes of MWCNTs were destroyed, the inner tubes were released to the contact areas, providing more new solid lubricants. On the test surface perpendicular to the drawing direction, MWCNTs can only be radially cut point by point, releasing limited carbon solid lubricants.

## Figures and Tables

**Table 1:** Formula and conditions for electroless Ni plating.

NiSO_4_·6H_2_O	40 (g/L)
C_6_H_5_Na_3_O_7_	60 (g/L)
NaH_2_PO_2_	20 (g/L)
NH_4_Cl	35 (g/L)
Temperature (°C)	20
pH	8.0

**Table 2:** Formula and conditions for electroless Cu plating.

CuSO_4_·5H_2_O	7.5 (g/L)
C_2_H_2_O_3_	27.5 (g/L)
EDTANa_2_·2H_2_O	89.4 (g/L)
2,2-Dipyridyl	0.01 (g/L)
Temperature (°C)	20
pH	11.5

## Data Availability

The data presented in this study are available on request from the corresponding author.
